# Electromagnetic navigation-guided posterior hemivertebra resection in adult congenital spinal deformity

**DOI:** 10.1093/jscr/rjaf870

**Published:** 2025-11-06

**Authors:** Peter Brumat, Jure Leban, Igor Potparić, Miha Vodičar

**Affiliations:** Department of Spinal Surgery, Oxford University Hospitals NHS Foundation Trust, Oxford, United Kingdom; Faculty of Medicine, University of Ljubljana, Ljubljana, Slovenia; Spine Unit, Department of Orthopaedic Surgery, University Medical Centre Ljubljana, Ljubljana, Slovenia; Spine Unit, Department of Orthopaedic Surgery, University Medical Centre Ljubljana, Ljubljana, Slovenia; Faculty of Medicine, University of Ljubljana, Ljubljana, Slovenia; Spine Unit, Department of Orthopaedic Surgery, University Medical Centre Ljubljana, Ljubljana, Slovenia

**Keywords:** navigation, hemivertebra, congenital, scoliosis, spinal deformity

## Abstract

Hemivertebra is a congenital spinal anomaly often associated with unpredictable progression of spinal deformity, for which early conservative or surgical intervention is generally recommended. While well-documented in pediatric populations, literature on the management of symptomatic adult congenital spinal deformity remains limited. We report two consecutive cases of adult congenital scoliosis caused by an L3 hemivertebra, both successfully treated with one-stage, electromagnetic navigation (EMN)-guided posterior total hemivertebra resection and instrumented spinal fusion. EMN-guided posterior total hemivertebra resection offers a safe and accurate solution for managing symptomatic adult congenital scoliosis, while also minimizing intraoperative radiation exposure. This technique highlights its clinical utility in achieving precise resection, as evidenced by the two presented cases.

## Introduction

Hemivertebra is a congenital spinal anomaly with often unpredictable deformity progression [1]. Although its consequences, recommended early intervention, and different treatment modalities are well-documented in children, detailed case reports and specific techniques for managing this pathology in symptomatic adults remain scarce [2]. The benefits of electromagnetic navigation (EMN) in musculoskeletal and spinal surgery are recognized, enhancing accuracy, and safety, while eliminating intraoperative radiation exposure [3, 4]. We report a 3D real-time EMN-guided posterior total resection of a L3 hemivertebra in two consecutive adult females with congenital scoliosis.

## Case reports

### Case 1

**Figure 1:**
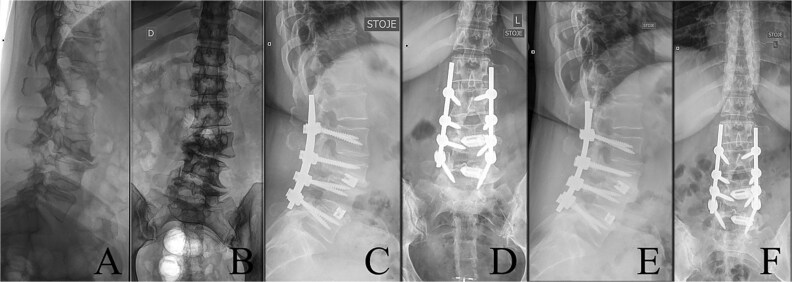
Last preoperative X-ray, lateral (A) and anteroposterior view (B). After 10 weeks of follow-up; lateral (C) and anteroposterior view (D). After 1 year of follow-up; lateral (E) and anteroposterior view (F).

**Figure 2:**
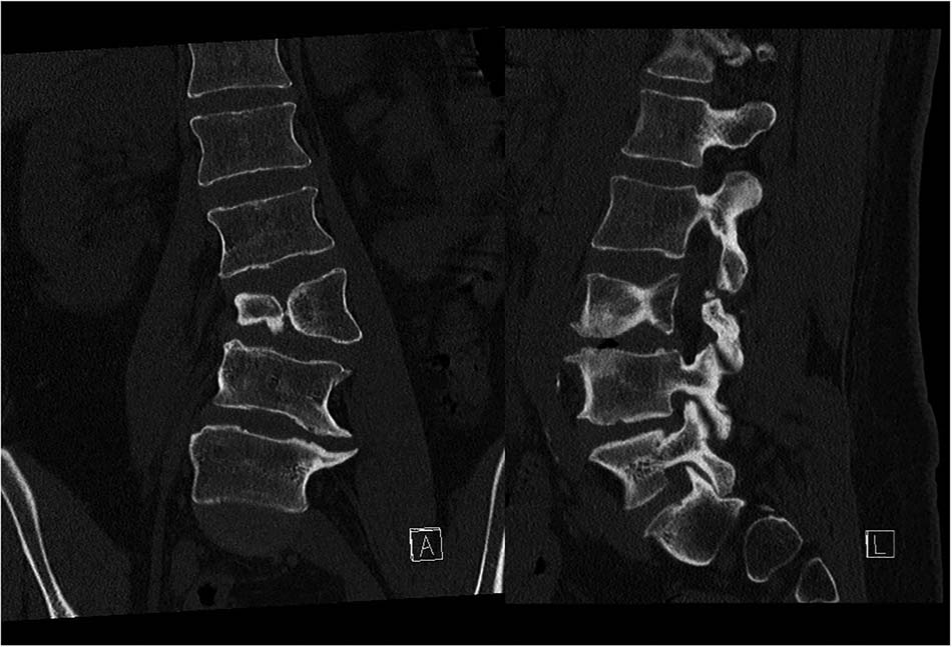
Preoperative CT demonstrating L3 hemivertebra; anteroposterior view (A) on the left side of the image, and lateral view (L) on the right side of the image.

A 43-year-old female with a history of a previous isolated right-sided L3 foraminotomy at an external facility (this intervention temporarily alleviated her leg pain but exacerbated her back pain) was referred to our institution due to chronic back pain, right-sided leg pain without neurological dysfunction, and a forward-leaning posture, which was correctable with verbal cues. The back pain, in the absence of neurological deficits, began 13 years prior, at which time she was diagnosed with adult congenital scoliosis caused by L3 hemivertebrae (Figs 1 and 2). Imaging confirmed a L3 hemivertebra resulting in a 24° Cobb angle of congenital scoliosis (Fig. 1), without associated cord abnormalities on MRI.

**Figure 3:**
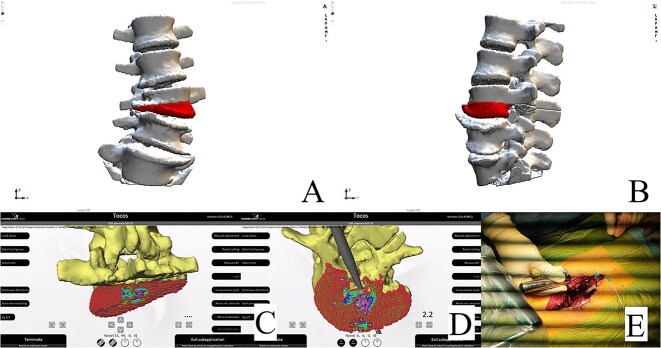
Resection plan, anteroposterior view (A). Resection plan, lateral view (B). Posterior and underside view of real-time intraoperative control of our preoperative plan; colored dots represent the planned resection area (C and D). Intraoperative check of the resection with the navigated probe (E).

CT based resection was 3D planned using EBS software (Ekliptik d.o.o., Ljubljana, Slovenia) (Fig. 3) [3]. After a standard open approach, pedicle screws were inserted from L2 to L5 under fluoroscopy control, followed by an L3 and L4 laminectomy and bilateral foraminotomy from L3 to L5. After removing right-sided adhesions, we entered the L3–L4 disc space and performed a 3D real-time EMN-guided (Guiding Star, Ekliptik d.o.o., Ljubljana, Slovenia) total resection of the L3 hemivertebra, following direct intraoperative control of our preoperative plan (Fig. 3) [3]. Fusion of the L3–L4 and L4–L5 segments was performed using a cage, augmented with autogenous bone grafting. Deformity correction was subsequently achieved using standard deformity correction maneuvers.

The postoperative course was uneventful, with no evidence of neurological dysfunction. The patient commenced physiotherapy on the first postoperative day. At the initial follow-up, 10 weeks post-surgery, she was ambulating unassisted with reduced back and leg pain, without neurological impairment but with a persistent forward-leaning posture that remained correctable with verbal cues. At the final follow-up, one year after surgery, she reported resurgence of forward-leaning posture after longer walks, and referred to a tertiary rehabilitation institution. Imaging demonstrated L2–L5 fusion with no signs of instability or residual deformity (Fig. 1).

### Case 2

**Figure 4:**
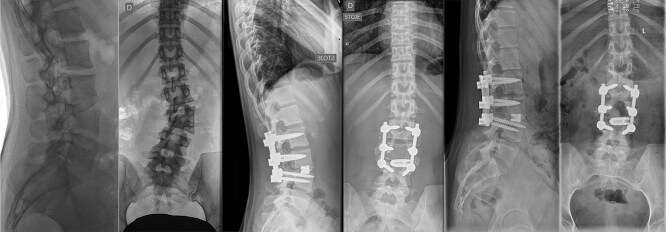
Last preoperative X-ray, lateral (A) and anteroposterior view (B). After 10 weeks of follow-up; lateral (C) and anteroposterior view (D). After 1 year of follow-up; lateral (E) and anteroposterior view (F).

**Figure 5:**
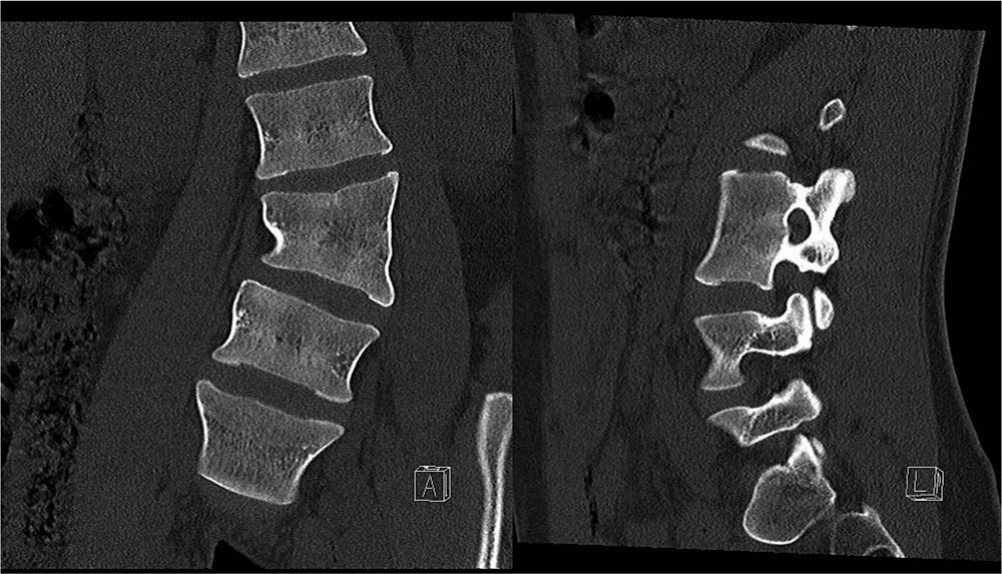
Preoperative CT demonstrating L3 hemivertebra; anteroposterior view (A) on the left side of the image, and lateral view (L) on the right side of the image.

A 24-year-old female with congenital scoliosis caused by a semi-segmented hemivertebra at L3 (Figs 4 and 5) was under follow-up at our institution for chronic back pain and left leg pain, without motor deficits. She had been referred to our institution three years earlier, at which time surgical resection was indicated. However, the procedure was postponed due to patient's personal reasons.

**Figure 6:**
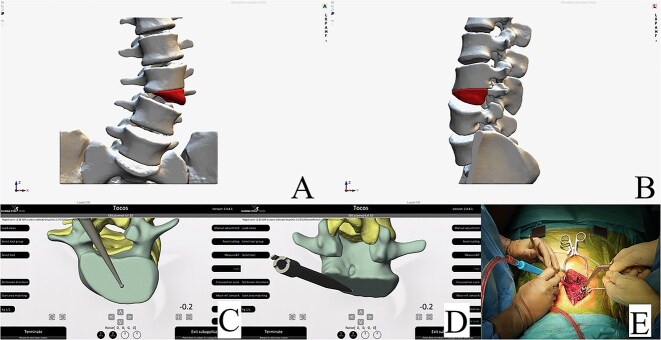
Resection plan, anteroposterior view (A). Resection plan, lateral view (B). Underside and posterior view of real-time intraoperative control of our preoperative plan; resection margin check with the navigated probe (C and D). Intraoperative view of the posterior hemivertebra resection (E).

CT-based 3D reconstruction revealed a semi-segmented hemivertebra at L3, resulting in a 32° Cobb angle of congenital scoliosis (Fig. 4). We decided to perform hemivertebra resection, L3 laminectomy, L2-L4 fixation with L3–4 cage fusion, and scoliosis correction. CT-based resection was 3D planned using EBS software (Fig. 6). Fixation from L2 to L4 was achieved using pedicle screws under fluoroscopic guidance, followed by L3 laminectomy. After accessing the L3–L4 disc space, a 3D real-time EMN-guided total resection of the L3 hemivertebra was performed (Fig. 6), and a cage with autogenous bone graft was inserted at the L3–L4 level. The left-sided L3 nerve was found to have an accessory branch, which was successfully preserved. Scoliosis was corrected using standard deformity correction maneuvers.

Postoperatively, the patient experienced left psoas and quadriceps femoris muscle paresis, with the latter gradually improved to near-normal before discharge. No other complications were observed. At the 10-week follow-up, the left quadriceps had regained full strength, but partial paresis (3 out of 5) of the psoas muscle persisted, resulting in gait disturbance. At the final follow-up, one year after surgery, imaging showed L2-L4 fusion with no signs of instability or residual deformity (Fig. 4). The patient’s back pain resolved. A normal muscle function was observed.

## Discussion

Research on characteristics and treatment modalities of adult congenital spine deformity remain scarce [2]. Adults with undiagnosed or late diagnosed hemivertebra can become symptomatic and may need surgical treatment, whereby for congenital anomalies early intervention may be suggested [5]. As demonstrated in our two reported cases, adult congenital deformity patients—primarily young females with hemivertebra—typically pursue surgery to address pain, functional impairment, and aesthetic concerns, rather than solely based on radiographic deformity [2].

Posterior resection with transpedicular fixation and short fusion seems safe and effective surgical treatment in carefully selected pediatric and adult cases despite potential complications, with CT-based computer-assisted techniques further enhancing precision and safety [1, 6, 7]. However, anatomical and biomechanical differences between adult and pediatric spines may influence the effectiveness of these established techniques. Restoring appropriate segmental sagittal alignment is paramount in adults, as spinal aging with disc height loss may result in kyphotic angulation at the site of the hemivertebra [7]. Column reconstruction with a cage may offer better deformity correction, sagittal balance, and fewer complications than posterior-only hemivertebra resection [8]. Khan *et al*. [9] reported on a successful two-stage approach for lumbar hemivertebra resection in an adult, using transpedicular osteotomy, multilevel posterior fixation and anterior interbody fusions using interbody cages, and bone morphogenetic protein. We, in both cases, opted for a one-stage posterior EMN-guided total L3 hemivertebra resection based on our prior experience with the resection of a recurrent sacral osteoblastoma, where, besides improving safety and accuracy, EMN also facilitated precise lesion localization and tailored resection without additional intraoperative radiation [3]. Similarly, Fisahn *et al*. [10] reported enhanced surgical accuracy and reduced radiation exposure with the use of cone-beam navigation for lumbar hemivertebra resection in congenital scoliosis.

## Conclusions

EMN-guided posterior hemivertebra resection offers a safe and accurate solution for managing adult congenital scoliosis, minimizing intraoperative radiation exposure. Additionally, this technique demonstrates its utility in achieving precise resection, as evidenced by the two presented cases.

## Informed consent statement

The patients provided written informed consent for their participation in the study and for their anonymized data to be published in this article.

## Data Availability

This article presents a case report and therefore the data are not available publicly or upon request to protect the privacy and identity of the patients.
